# Initial Evaluation of Pain Intensity Among Depressed Patients as a Possible Mediator Between Depression and Pain Complaints

**DOI:** 10.3389/fpsyt.2019.00048

**Published:** 2019-02-15

**Authors:** Uri Nitzan, Maya Hecht, Yoram Braw, Hagai Maoz, Yechiel Levkovitz, David Yarnitsky, Yelena Granovsky, Yuval Bloch

**Affiliations:** ^1^Shalvata Mental Health Center Hod-Hasharon, Israel; ^2^Sackler School of Medicine, Tel-Aviv University, Tel-Aviv, Israel; ^3^Department of Psychology, Ariel University, Ariel, Israel; ^4^Be'er Yaakov Mental Health Center, Be'er Yaakov, Israel; ^5^Department of Neurology, Rambam Medical Center, Haifa, Israel; ^6^Technion Faculty of Medicine, Haifa, Israel

**Keywords:** depression, pain, conditioned-pain-modulation (CPM), pain-processing, pain-catastrophizing

## Abstract

Pain complaints are frequently described by depressed patients, and are mostly attributed to abnormal pain perception and modulation. The present study aimed to assess whether a unique pain processing profile differentiates depressed patients from healthy controls. Participants were 25 patients suffering from a moderate-severe unipolar depressive episode and 25 age and sex-matched healthy controls. Thermal stimuli were used to assess sensory threshold and pain threshold. Pain-60 temperature (temperature that induces pain ratings of 60 out of 100) was the first noxious stimuli to be administered during the experimental session. Central pain inhibition was assessed via conditioned pain modulation (CPM) and the degree of central nervous system excitability was assessed via mechanical temporal summation. Depressed patients reported higher levels of pain compared with healthy controls, and a significantly higher perceived pain during the last month. Additionally, they displayed significantly lower pain-60 temperature values compared with healthy controls (*p* = 0.01). Otherwise, no significant group differences were found in measures of pain perception and modulation. Our results suggest that the initial evaluation of pain intensity among depressed patients, as validated by pain-60 temperature values, is increased compared with healthy controls, and might be the mediator between depression and pain complaints. Possibly, depressed patients' negative bias in the processing of pain is similar to their processing pattern of facial expression or future events. Further studies are necessary in order to establish the mechanisms underlying the excessive pain complaints reported by patients with unipolar depression.

## Introduction

Pain symptoms are extremely common in depressed patients (1), and up to 80% of patients who present in primary care settings with major depression suffer from physical symptoms such as pain (2)_._ A significant line of evidence shows increased thresholds for experimentally-induced pain (i.e., reduced perception of phasic cutaneous heat pain) in patients with major depressive disorder (3, 4). In other words, the “paradox of pain” in depressed individuals is manifested in decreased experimentally-induced pain sensitivity combined with a high incidence of reported endogenous pain (5). In an attempt to explain this paradox, Ward et al hypothesized that different parts of the spinothalamic tract are involved in the processing of experimental noxious stimuli vs. those that are involved in clinical pain (6). At the core of additional hypothesis in the literature (7, 8) is the assumption that a deficit in central pain inhibition plays an essential role in the pathophysiology of pain symptoms in depression and might explain their high incidence. Nonetheless, prior studies (3, 9) did not support this assumption, and failed to demonstrate decreased central pain inhibition among depressed patients when compared to healthy controls (HC). An alternative explanation is that the excessive pain symptoms among depressed patients are a result of the patients' negative evaluation-bias in processing complex sensory input, just as is their processing pattern of facial expression and rating of future events (10).

The present study assessed the hypothesis that a unique pain processing profile typifies depressed patients and is correlated with a high incidence of pain symptoms in unipolar depression. Thus, we utilize a gold standard quantitative measure of pain processing in order to assess whether the latter is affected by a negative bias in depressed patients.

## Materials and Methods

### Study Population

Depressed patients were recruited from the open ward, day admission, and outpatient clinics at the Shalvata Mental Health Center, Hod-Hasharon, Israel. Control subjects (HC) were mostly recruited from the hospital staff. All participants provided written informed consent to participate in a protocol approved by the local IRB. Participants were required to have a DSM 5 diagnosis of Major Depressive Disorder and currently be experiencing a major depressive episode (HDRS-21 total score ≥ 9). Exclusion criteria were as follows: psychotic spectrum disorders or bipolar disorder (DSM-IV criteria); chronic pain conditions or rheumatic disorders; pregnancy or lactation; agitation; drug/alcohol use 48 h prior to the study. All depressed patients were prescribed medications during the study. Most of them received a mixed regimen of antidepressants and at least one additional antidepressant, anxiolytic, or an antipsychotic/mood-stabilizer augmentation therapy (*N* = 17, 68%). Seven patients (28%) were using other psychotropic medications, without an antidepressant, at the time of the study.

### Procedure

All participants completed a single 3-h session. Participants completed questionnaires including demographic and clinical information, as well as undergoing a thorough clinical evaluation of their emotional and cognitive status by way of an interview and the following scales: the Hamilton Anxiety Rating Scale (HAM-A); the Mini-Mental State Examination (MMSE); the Hamilton Depression Rating scale (HDRS), the Quick Inventory of Depressive Symptoms (QIDS-SR16), and the Clinical Global Impressions (CGI) Scale to assess depression severity; and finally the Brief Symptom Inventory (BSI) and the Brief Pain Inventory (BPI) to evaluate pain symptoms and the subjective experience of pain. HAM-A, HAM-D, MMSE, and the CGI were administered by members of the study group who were qualified by an experienced senior psychiatrist. Inter-rater reliability was conducted between raters, but blinding or obligating a “minimum time of contact” was not possible in the context of the present study and its limitations. The clinical evaluation was followed by a psycho-physical test of pain parameters (11, 12).

TSA-II (Medoc, Ramat Yishai, Israel), a Peltier-based contact temperature stimulation device with a 30 × 30 mm^2^ contact thermode, was used to assess heat sensory threshold, heat pain threshold. Von Frey Filaments (North Coast Medical, San Jose, California) were used to determine mechanical temporal summation.

Pain-60 temperature is the temperature that induces pain ratings of 60 on a numerical pain scale of the participants' subjective pain experience from 0 (*no pain*) to 100 (*worst pain ever*). Subjects were exposed to a series of hot stimuli of 7 s duration. The first series consisted of 45, 46, and 47°C stimulations with a 1-min inter-stimulus interval. After each stimulus, subjects were asked to verbally report the level of pain, until the stimuli that induced *pain-*60 was detected and confirmed. Pain-60 was the first noxious stimuli to be administered during the experimental session, and is considered to reflect one's initial processing of pain and encoding of pain intensity. CPM is designed to assess the central pain inhibition ability of the participant via the “pain inhibits pain” paradigm. CPM was performed using the TSA-II and the parallel paradigm in which the identical noxious “test stimuli” is repeated twice; one delivered before to, and then simultaneously with, a noxious “conditioning stimulus.” See Appendix 1 for full details on stimulation protocol and an elaborate description of all psychophysical measures of pain.

### Data Analysis

Analyses were conducted using the IBM Statistical Package for Social Sciences (SPSS), Version 21. A two-tailed *p*<*0.0*5 was considered statistically significant for all comparisons.

Independent-samples *t*-tests were used to compare the groups in the following measures: pain-60 temperature, pain threshold, sensory threshold, mechanical temporal summation, and CPM. Hierarchical linear regressions were performed in order to achieve a better understanding of the factors affecting the perception of pain. More specifically, we analyzed whether depressive symptoms could predict perception of experimental pain after controlling for possible confounders and subjective perception of pain during the study (i.e., current BPI). Separate regressions were conducted for pain measures in which DEP patients and HC significantly differed (BSI total score and pain-60 temperature). Possible predictors were entered in three blocks: (1) Two variables in which the groups differed significantly, were suspected as possible confounders. More specifically, the groups differed in education level and MMSE total score, two variables that were found to be associated with pain in earlier studies (13, 14). They were, therefore, entered in the first block of the regression analyses, (2) Perceived pain during the study (i.e., current BPI), and (3) depressive symptoms (i.e., HDRS total score).

Multivariate analysis of variance (MANOVA) with a between-subjects factor of *group* (DEP/HC) was used for group comparison in the BPI sub-components (mean experienced pain, current pain, and functioning). Next, the groups were compared in BSI (total score), using an independent-samples *t*-test.

Since the groups differed in education level, MMSE total score, and pain-60 temperature, the analyses were repeated using these variables as covariates (i.e., the independent-samples *t*-tests and MANOVA comparing the groups in measures of pain). The findings were almost identical to those of the original analyses and are therefore not reported.

## Results

### Patient Characteristics

Study population included twenty-five adult patients (16 females, 64%) and 25 HC (13 females, 52%), matched in age and sex. Patients had an average HDRS-21 total score of 24.08 (±5.79), and 10 of them were in the severely depressed range (HDRS-21 total score >25). See Table 1 for broader demographic and clinical data.

**Table 1:** Demographic and clinical measures of depressed patients (*n* = 25) and healthy controls (*n* = 25).

**Measures**		**Depressed patients**	**Healthy controls**	**Statistical analyses**
Age (years) [Mean ± SD]		43.6 ± 14.7	37.7 ± 10.6	*t*_(48)_ = 1.62, *p* = 0.111.
Education level (no.) [Mean ± SD]		13.2 ± 4.0	17.1 ± 2.5	*t*_(47)_ = −3.98, *p* < 0.001
Gender (male/female) [Mean ± SD]		9/16	12/13	*X*${}_{\left(1\right)}^{2}$ = 0.74, *p* = 0.390
Age at first episode (years) [Mean ± SD]		29.3 ± 15.5	___	___
Number of depressive episodes (no.) [Mean ± SD]		3.8 ± 2.4	0.0 ± 0.0	*t*_(48)_ = 7.71, *p* < 0.001
Number of hospitalizations (no.) [Mean ± SD]		2.8 ± 2.4	0.0 ± 0.0	*t*_(48)_ = 5.83, *p* < 0.001
HAM-D total score (no.) [Mean ± SD]		24.1 ± 5.8	1.7 ± 2.2	*t*_(48)_ = 18.06, *p* < 0.001
HAM-A total score (no.) [Mean ± SD]		22.6 ± 7.1	2.3 ± 2.3	*t*_(48)_ = 13.52, *p* < 0.001
CGI (no.) [Mean ± SD]		5.2 ± 0.9	1.0 ± 0.0	*t*_(48)_ = 22.90, *p* < 0.001
BSI (no.) [Mean ± SD]		17.2 ± 11.3	3.0 ± 2.8	*t*_(47)_ = 6.10, *p* < 0.001
Psychiatric medications (no. [%])	Antidepressants (exclusively) [N (%)]	1 (4%)	___	___
	Mixed group^a^ [N (%)]	17 (68%)	___	___
	Without antidepressants [N (%)]	7 (28%)	___	___
MMSE total score [Mean ± SD]		28.2 ± 1.6	29.6 ± 0.6	*t*_(47)_ = −3.79, *p* < 0.001

*BSI, Brief Symptom Inventory; CGI, Clinical Global Impression; HAM-A, Hamilton Rating Scale for Anxiety; HAM-D, Hamilton Rating Scale for Depression; MMSE, Mini-Mental State Examination*.

a *Antidepressants with combination of neuroleptics and/or mood stabilizers and/or benzodiazepines*.

Depressed patients had significantly lower pain-60 temperature scores than the HC (*p* = 0.01), indicating that their appraisal of the intensity of pain was increased in comparison to the HC (see Table 2).

**Table 2:** Primary and secondary outcome measures of depressed patients (*n* = 25) and healthy controls (*n* = 25).

**Measures**		**Depressed patients (mean ± SD)**	**Healthy controls (mean ± SD)**	**Statistical analyses**
BPI	Mean-experienced pain (no.)	3.2 ± 2.5	1.9 ± 0.9	*F*_(1, 45)_ = 5.90, *p* = 0.019
	Current pain (no.)	2.9 ± 2.5	1.2 ± 0.7	*F*_(1, 45)_ = 9.55, *p* = 0.003
	Functioning (no.)	3.9 ± 2.7	1.3 ± 0.7	*F*_(1, 45)_ = 18.95, *p* < 0.001
BSI (total score)		17.2 ± 11.3	3.0 ± 2.8	*t*_(47)_ = 6.10, *p* < 0.001
Pain threshold (no.)		40.2 ± 2.4	40.1 ± 2.4	*t*_(48)_ = 0.08, *p* = 0.933
Sensory threshold (no.)		34.2 ± 1.0	33.8 ± 0.7	*t*_(48)_ = 1.66, *p* = 0.103
mTS (no.)		1.4 ± 2.4	1.6 ± 2.7	*t*_(46)_ = −0.17, *p* = 0.866
Final CPM (no.)		6.1 ± 21.9	6.2 ± 16.5	*t*_(48)_ = −0.02, *p* = 0.984
Pain-60 temperature (no.)		43.1 ± 3.2	45.2 ± 2.0	*t*_(48)_ = −2.68, *p* = 0.010
Bath temperature (°C)		44.18 ± 0.6	44.66 ± 0.50	*t*_(47)_ = −3.03, *p* = 0.004

*BPI, Brief Pain Inventory; BSI, Brief Symptom Inventory; CPM, Conditioned Pain Modulation; mTS, mechanical Temporal Summation*.

DEP patients had significantly higher scores on the BPI mean-experienced pain, and current functioning components, than did the HC, and a significantly higher BSI total scores (perceived pain during the last month). The groups did not significantly differ in the sensory threshold and pain threshold measures, as well as the mechanical temporal summation and CPM (See Table 2). No meaningful results were found in the linear regressions that were conducted.

No differences were found on any of the pain measures between patients taking antidepressants (exclusively or in conjunction with other medications) and patients taking other psychotropic medications. Follow-up ANOVAs indicated that patients with high HDRS-21 total scores had significantly higher scores on all BPI measures than did mildly depressed patients: BPI mean-experienced pain, *F*_(1, 19)_ = 4.68, *p* = 0.043; current BPI, *F*_(1, 19)_ = 10.19, *p* = 0.005; and BPI functioning, *F*_(1, 19)_ = 5.81, *p* = 0.026. Patients with higher HDRS-21 total scores also had significantly higher BSI total scores compared to DEP patients with fewer depressive symptoms, *t*_(19)_ = −3.00, *p* = 0.007.

## Discussion

Our findings support the hypothesis that a unique sensory-perceptual profile differentiates patients with depression from healthy controls. Depressed patients in our sample displayed lower pain-60 temperature values compared to those manifested by HC. Thus, compared with HC, a lower temperature (weaker experimental noxious heat stimuli) led depressed patients to experience pain-60 and evaluate it as such on the numerical pain scale. A theoretical explanation for this negative bias might be based on the central role of the primary somatosensory cortex (S1) and the thalamus in human central pain processing (15). S1 has been demonstrated to be the only region whose activation magnitude significantly predicted the subjective intensity coding of noxious stimuli and correlated with subjective pain ratings. Pain-60 represents the encoding of pain intensity, and the abnormal functional connectivity between S1 and the thalamus in depressed patients (16) might explain the lower pain-60 temperature values displayed by depressed patients.

As expected, pain-60 temperature was inversely correlated with all psychopathology measures in the study Thus, the initial evaluation of pain intensity, as validated by pain-60 temperature, seems to be central in the pathophysiology of pain in depression.

The fact that other parameters of pain sensation and modulation did not differ between the groups might be explained by the fact that the heat pain stimuli used to determine pain-60 temperature was the first noxious stimuli to be administered during the experimental session. It is likely that the response to additional heat stimuli administered later was influenced by the previous noxious stimuli and did not reflect a naïve representation of pain processing. Alternatively, it is the influence of depression on the attention span to the pain stimuli (17) that accounts for this effect. More specifically, full attention is given to the initial pain stimuli (hypersensitivity), and as the participant progresses through the study protocol his/hers attention decreases and so does his sensitivity to pain. This is further supported by studies which tried to categorize depressed patients as having an affective indifference to experimental aversive stimulation, or a stoic pain behavior that underlay their putative pain insensitivity (18, 19)_._ Hence, we suggest that it is the pain-60 temperature that represents the unperturbed pain processing pattern in our study population.

Pain catastrophizing has emerged as one of the most robust, and reliable, psychological predictors of pain experience (20). Possibly, depressed patients' increased evaluation of pain intensity accords with their cognitive bias, and is similar to their processing pattern of facial expression; consistent evidence demonstrates that individuals suffering from depression have a negative response bias toward sadness, so that they tend to evaluate positive (happy), neutral, or ambiguous facial expressions as sadder or less happy than do HC (11, 21). Combined with our results regarding pain-60 temperature, factors such as pain catastrophizing might be responsible for the cognitive-emotional bias and high incidence of pain symptoms in depressed individuals.

In our study no significant difference in terms of pain threshold and central pain inhibition but analyzing the literature and the possible reasons for that is beyond the scope of this paper.

A considerable limitation of the study is that Pain-60 is not an established test for evaluation bias, and that we did not use other tests for evaluation biases. Nonetheless, we do think that the measure of pain-50 or pain-60 temperature can potentially serve as a test for higher pain responsiveness; a recent study of our collaborators (22) demonstrated a lower Pain-50 temperature in patients with mild traumatic brain injury pain as compared with healthy controls. Thus, we believe that our finding on Pain-60 temperature as differentiating between the pain sensory-perceptual profile of patients suffering from major depression and healthy controls is not surprising.

Antidepressant medications (AD) might have an anti-nociceptive effect, and most depressed patients in our study were taking various regimens of antidepressants during the trial. Nonetheless, in a previous study, Bar et al. (9) could not show any influence of antidepressants on pain thresholds in depressed patients. Additionally, the type of pain stimulation applied can influence the results of a study such as this one, as can the physical and mental comorbidity among the study population. Finally, in the context of a brief report we could not discuss the important implications of gender differences on pain perception and modulation (23).

The present study stresses that the initial evaluation of pain intensity among depressed patients is increased compared with HC. This negative bias might affect the way pain is experienced by depressed patients and serve as a mediator between depression and pain complaints. Further studies are needed to unravel the mechanisms underlying the excessive pain symptoms reported by depressed patients.

## Author Contributions

UN, DY, and YG designed the study and wrote the protocol. UN and YL managed the literature searches and wrote the first draft of the paper. YoB and HM helped in designing the study, undertook the statistical analysis, and edited the first draft of the paper. MH and YuB managed and conducted the study and edited the first draft of the paper. All authors contributed to and have approved the final manuscript.

### Conflict of Interest Statement

The authors declare that the research was conducted in the absence of any commercial or financial relationships that could be construed as a potential conflict of interest.

## References

[1] Kirmayer LJ Robbins JM Dworkind M Yaffe MJ. Somatization and the recognition of depression and anxiety in primary care. Am J Psychiatry (1993) 150:734–41. 10.1176/ajp.150.5.734 8480818

[2] Stahl SM. Does depression hurt? J Clin Psychiatry (2002) 63:273–74. 10.4088/JCP.v63n0401 12000200

[3] Normand E Potvin S Gaumond I Cloutier G Corbin JF Marchand S. Pain inhibition is deficient in chronic widespread pain but normal in major depressive disorder. J Clin Psychiatry (2011) 72:219–24. 10.4088/JCP.08m04969blu 20816025

[4] Suzuki R Rygh LJ Dickenson AH. Bad news from the brain: descending 5-HT pathways that control spinal pain processing. Trends Pharmacol Sci. (2004) 25:613–7. 10.1016/j.tips.2004.10.002 15530638

[5] Bär KJ Terhaar J Boettger MK Boettger S Berger S Weiss T. Pseudohypoalgesia on the skin: a novel view on the paradox of pain perception in depression. J Clin Psychopharmacol. (2011) 31:103–7. 10.1097/JCP.0b013e3182046797 21192152

[6] Ward NG Bloom VL Dworkin S Fawcett J Narasimhachari N Friedel RO. Psychobiological markers in coexisting pain and depression: toward a unified theory. J Clin Psychiatry (1982) 43:32–9. 6178726

[7] Lautenbacher S Krieg JC. Pain perception in psychiatric disorders: a review of the literature. J Psych Res. (1994) 28:109–22. 10.1016/0022-3956(94)90023-X 7932274

[8] Bär KJ Brehm S Boettger MK Boettger S Wagner G Sauer H. Pain perception in major depression depends on pain modality. Pain (2005) 117:97–103. 10.1016/j.pain.2005.05.016 16061323

[9] Bär KJ Greiner W Letsch A Köbele R Sauer H. Influence of gender and hemispheric lateralization on heat pain perception in major depression. J Psych Res. (2003) 37:345–53. 10.1016/S0022-3956(03)00051-7 12765857

[10] Bourke C Douglas K Porter R. Processing of facial emotion expression in major depression: a review. Aust N Z J Psychiatry (2010) 44:681–96. 10.3109/00048674.2010.496359 20636189

[11] Yarnitsky D Crispel Y Eisenberg E Granovsky Y Ben-Nun A Sprecher E. Prediction of chronic post-operative pain: pre-perative DNIC testing identifies patients at risk. Pain (2008)138:22–8. 10.1016/j.pain.2007.10.033 18079062

[12] Nir RR Granovsky Y Yarnitsky D Sprecher E Granot MA. Psychophysical study of endogenous analgesia: the role of the conditioning pain in the induction and magnitude of conditioned pain modulation. Eur J Pain (2011) 15:491–7. 10.1016/j.ejpain.2010.10.001 21035364

[13] Jiang X Sandberg ME Saevarsdottir S Klareskog L Alfredsson L Bengtsson C Higher education is associated with a better rheumatoid arthritis outcome concerning for pain and function but not disease activity: results from the EIRA cohort and Swedish rheumatology register. Arthritis Res Ther. (2015) 17:317 10.1186/s13075-015-0836-6 26546562 PMC4636760

[14] Pickering G Jourdan D Eschalier A Dubray C. Impact of age, gender and cognitive functioning on pain perception. Gerontology (2002) 48:112–8. 10.1159/000048937 11867935

[15] Fields H. Pain and the primary somatosensory cortex. Pain (2012) 153:742–3. 10.1016/j.pain.2012.01.034 22365311

[16] Canavero S Bonicalzi V. Role of primary somatosensory cortex in the coding of pain. Pain (2013) 154:1156–59. 10.1016/j.pain.2013.02.032 23590938

[17] Kuner R. Central mechanisms of pathological pain. Nat Med. (2010) 16:1258–67. 10.1038/nm.2231 20948531

[18] Hall KRL Stride E. The varying response to pain in psychiatric disorders: a study in abnormal psychology. Psychol Psychother. (1954) 27:48–60. 10.1111/j.2044-8341.1954.tb00848.x 13160300

[19] Davis GC Buchsbaum MS Bunney WE. Analgesia to painful stimuli in affective illness. Am J Psychiatry (1979) 136:1148–51. 10.1176/ajp.136.9.1148 474801

[20] Gatchel RJ Peng YB Peters ML Fuchs PN Turk DC. The biopsychosocial approach to chronic pain: scientific advances and future directions. Psychol Bull. (2007) 133:261–71. 10.1037/0033-2909.133.4.581 17592957

[21] Smeets RJ Vlaeyen JW Kester AD Knottnerus JA. Reduction of pain catastrophizing mediates the outcome of both physical and cognitive-behavioral treatment in chronic low back pain. J Pain (2006) 7:261–27. 10.1016/j.jpain.2005.10.011 16618470

[22] Kuperman P Granovsky Y Granot M Bahouth H Fadel S Hyams G. Psychophysic-psychological dichotomy in very early acute mTBI pain: a prospective study. Neurology (2018) 91:e931–8. 10.1212/WNL.0000000000006120 30068635

[23] Belfer I. Pain in women. Agri (2017) 29:51–4. 10.5505/agri.2017.87369 28895988

