# Supplementary material for: Initial Evaluation of Pain Intensity Among Depressed Patients as a Possible Mediator Between Depression and Pain Complaints
Source: Front Psychiatry. 2019 Feb 15;10:48. doi: 10.3389/fpsyt.2019.00048 (PMC6384224; doi:10.3389/fpsyt.2019.00048)
Supplement: Supplementary file 1 [file Data_Sheet_1.PDF]

Appendix 1

Psychophysical assessments[1-3]

All pain psychophysical tests were performed at the volar aspect of the dominant forearm.

1. **Sensory threshold** was assessed by the method of limits. First, the thermode was attached and the skin was allowed to adapt to a temperature of 32_C. For the sensory threshold assessment, the thermode warmed or cooled at a slow rate (1_C/s) until the first warm sensation pain was perceived.
2. For the **heat pain threshold**, the temperature was increased at a rate of 2_C/s until the moment that the subject indicated the transition of the warm sensation into a painful heat. Each test was repeated 3 times, and the results were averaged to obtain the threshold value.
3. Conditioned Pain Modulation (CPM) is the psychophysical paradigm used to test central pain inhibition. It was performed using the parallel paradigm in which 2 identical noxious 'test stimuli' were delivered before to, and then simultaneously with, a noxious 'conditioning stimulus.' Contact heat applied to the volar forearm served as the 'test stimulus.' The intensity of the test stimulus was predetermined individually for each participant, based on the psychophysical parameter of pain-60, which is the temperature that induces pain ratings of 60 on an NPS of 0 to 100. The baseline temperature was 32_C, with increase and decrease rates of 2_C/s. Patients were asked to rate the level of pain intensity of the 'test stimulus' every 10 seconds (overall 4 pain reports). After a 15-minute break, the non-dominant hand was immersed in the hot water bath (up to the wrist) at 46.5_C for 60 seconds. During the last 30

seconds of this immersion, the ‘test stimulus’ was repeated, and the level of pain intensity experienced was rated again

4. **Mechanical Temporal Summation** (mTS) is a psychophysical measure thought to reflect the degree of central nervous system excitability to noxious stimulation, a phenomenon referred to as central sensitization. It was measured using the 180gr (# 6.45) von Frey filament. Patients were exposed to a single stimulus and then to 10 repetitive stimuli with an inter-stimulus interval (ISI) of 1 second applied within an area 1 cm in diameter. They were asked to rate the level of pinprick pain intensity using NPS for the single stimulus and then for the last stimulus of the train. The difference between the last and the first pain scores was calculated as the mTS score.
5. **Pain-60**[4] is the temperature that induces pain experience at a magnitude of 60 on a 0–100 NPS (numerical pain scale). In general, subjects were exposed to a series of hot stimuli of 7 s duration. The first series consisted of 45, 46, and 47 _C stimulations with a 1-min inter-stimulus interval. After each stimulus, subjects were asked to verbally report the level of pain. If one of these stimuli induced pain- 60, that temperature was chosen as the test stimuli for the rest of the experiment; if not, additional steps for determination of pain-60 were applied. In order to reconfirm the pain-60 temperature, an additional stimulus at the same intensity was given and scored at the end of the process.

[1] Yarnitsky D, Crispel Y, Eisenberg E, Granovsky Y, Ben-Nun A, Sprecher E, et al. Prediction of chronic post-operative pain: pre-perative DNIC testing identifies patients at risk. Pain (2008)138: 22-8.

[2] Nir RR, Granovsky Y, Yarnitsky D, Sprecher E, Granot MA. psychophysical study of endogenous analgesia: the role of the conditioning pain in the induction and magnitude of conditioned pain modulation. Eur J Pain (2011) 15:491-7.

[3] Yarnitsky D, Granot M, Nahman-Averbuch H, Khamaisi M, Granovsky Y. Conditioned pain modulation predicts duloxetine efficacy in painful diabetic neuropathy. Pain 2012; 153(6):1193-8

[4] Granot M, Weissman-Fogel I, Crispel Y, Pud D, Granovsky Y, Sprecher E, Yarnitsky D. Determinants of endogenous analgesia magnitude in a diffuse noxious inhibitory control (DNIC) paradigm: Do conditioning stimulus painfulness, gender and personality variables matter? Pain 2008; 136:142–149.
